# Edward Fawcett

**Published:** 1942

**Authors:** 


### Obituary

### EDWARD FAWCETT, M.D. (Edin.), F.R.S.

On September 21st, Professor Fawcett, who for forty-one years held the Chair of Anatomy in the University College, and University of Bristol, died suddenly while walking in the street. He had occupied a pre-eminent place in the work of the University and in the affections of many generations of medical students. He came to Bristol as a young man in 1893, four years after he had qualified at Edinburgh University, to be the first full-time Professor of Anatomy in University College, and he captured the attention of a none too orderly crowd of medical students immediately by his capacity to lecture, to demonstrate, to illustrate by drawings and by his power of keeping order. Perhaps he had other gifts besides his grasp of anatomy that gave him authority in his class-room. He was an all-round athlete, and as a cricketer might have found a place in almost any county team. Edward Fawcett was born in 1867, the son of Thomas Fawcett, B.A., at Blencow, near Penrith, and was educated at the Grammar School there and at Edinburgh University. By 1887 he had become a demonstrator of Anatomy in the Edinburgh School of Medicine and after he graduated in 1889 he was demonstrator of anatomy at Yorkshire College, Leeds, till he was called to Bristol. In 1906 he became Dean of the Faculty of Medicine and he played an important part in organizing the Faculty when the University was given its charter three years later. The Vice-Chancellor (Dr. T. Loveday) writes the following appreciation and tribute: "As a colleague in administration Edward Fawcett was of a metal that always rang true. He had his prejudices, as who has not? But he recognized them as such and allowed for them himself, so that his view of a problem was objective, fair and generous. His opinions were well considered and fortified by long experience accurately remembered. They were clear-cut and he expressed them forcibly, though never clamourously; if, rarely, they were not accepted, he co-operated with full energy in the policy adopted. He was entirely loyal to the University which he served, always helpful, always dependable. He was upright-fashioned and four-square, solid, strong and firm." For he was able and diligent in his attention to affairs. Academic honours naturally came to him, and in 1906 he won the gold medal for his M.D. thesis at Edinburgh. In 1909 he was made President of the Anatomical

Section of the International Congress of Medicine at Budapest, and from 1927 to 1929 he also held that office in the Anatomical Society.

Fawcett's researches into the development of the human skeleton and of the mammalian chrondrocranium won for him the F.R.S. in 1923, but the beautiful wax models which illustrated his work have been lost by enemy action. Apart from his prowess at cricket Fawcett had many hobbies. He was an indefatigable cyclist and used his bicycle on many expeditions to study church architecture. He was a first-rate photographer, and some of his best work illustrated his Presidential Address to the Bristol and Gloucestershire Archæological Society in 1936, when he spoke on anthropophagous sculpture and royal arms in churches. For many years he represented Bristol on the Central Council for the Preservation of Churches, and for the past months he had been re-photographing local churches for the National collection. Prehistory was another of his recreations, and he was energetic in his support of the University Spelæological Society, of which he was for many years President, and he was the enthusiastic comrade of the youngest undergraduates on expeditions to explore the caves of the Mendips.

Professor Fawcett married, in 1895, Edith Dora Wordsworth, and he leaves her with a daughter and a son, A. W. Fawcett, surgeon to the Sheffield Royal Infirmary.

